# Editorial: putting patients first: revolutionizing mental health care

**DOI:** 10.1093/eurpub/ckae078

**Published:** 2024-06-07

**Authors:** Charlotte Marchandise, Stella Kyriakides, Hans Henri Kluge, Ricardo Mexia, Floris Barnhoorn

**Affiliations:** EUPHA Executive Director; European Commissioner for Health and Food Safety; WHO/Europe Regional Director; Chair of the 17th EPH Conference, Lisbon 2024; Deputy Director EPH Conference

Dear Readers,

Innovation in healthcare is a theme that we will particularly navigate this year, as it is the theme of our next EPH conference. At the European Public Health Association, we are looking forward to questioning innovation as advocates for evidence-based policy, as well as through all our Sections’ points of view. We are also dedicated to champion for innovation that genuinely places patients at the core of healthcare decisions.

In this piece, I would like to share a personal experience. In the realm of mental health treatment, despite significant advancements in medical science, the journey to identifying the most effective treatment for mental health conditions remains fraught with trial and error, especially when it comes to psychopharmacology. The traditional approach of sequentially trying different medications can be a lengthy, sometimes debilitating process for patients. It is here that the potential for innovation, specifically through genetic blood testing, presents hope.

Imagine a scenario where, instead of navigating through an odyssey of pharmaceutical trials, we could utilize precision medicine to pinpoint the most effective treatment from the outset. Blood tests that analyse genetic markers can provide critical insights into how individuals metabolize various medications, including antidepressants. This capability is not just a theoretical possibility but a practical innovation that can significantly change the landscape of mental health treatment.

The utility of such an approach extends beyond just the immediate benefits to individual patients. Adopting genetic testing for medication metabolism promises a more cost-effective healthcare system by diminishing the need for repeated consultations, reducing both the physical and psychological impacts of ineffective treatments, and shortening the time required to find the appropriate medication.

Sharing from personal experience, after 3 terrible years and over 25 different medication trials for my child, a genetic test revealed a rapid metabolism for specific drugs. This revelation could have spared years of trial and error, highlighting the urgent need for integrating genetic testing into the standard protocol for prescribing psychiatric medications. These years were—and are—also years of suffering, with a huge deterioration of trust and physical health, and a continuous feeling of failure. As my kid says, ‘it’s like doctors never believed that the medicine did not work for me, as if it was my fault, until finally, the science proved me right. And now, what?’

Our approach to mental health treatment must harness innovation, for and with the patients, to ensure that healthcare becomes more personalized, effective and compassionate. Acknowledging the costs involved, the investment in such technologies is dwarfed by the expenses associated with ongoing therapeutic misadventures.

This discourse necessitates a confluence of ethics, science, policymakers, healthcare providers and the wider public health community to embrace innovative solutions capable of transforming the lives of those contending with mental health conditions.

Innovation in healthcare transcends mere technological advancements; it is about fundamentally reorienting our perspective to prioritize every patient’s well-being and dignity. Innovation should pave the way to a future where every individual has access to the right treatment at the right time, treating them as whole beings rather than as collections of isolated pathologies.

Recent studies, such as the one conducted by Cellini et al. (Cellini L, De Donatis D, Zernig G, *et al*. Antidepressant efficacy is correlated with plasma levels: mega-analysis and further evidence. *Int Clin Psychopharmacol*. 2022;37:29–37), published in the *International Clinical Psychopharmacology* in 2022, spotlight a critical advancement in our understanding of antidepressant efficacy. Demonstrating a clear correlation between antidepressant plasma levels and their effectiveness, this research bolsters the argument for therapeutic drug monitoring as an essential tool in optimizing patient care. This leap forward in personalized medicine not only promises to enhance treatment outcomes but also underscores the need for swift integration of scientific discoveries into clinical practice.

WHO Europe estimates that three out of four people with major depression do not receive adequate treatments. This is a huge number that we should address as a science-based organization, and as society. Innovation in healthcare transcends mere technological breakthroughs; it requires our collective vision to reorient healthcare systems to prioritize the well-being and dignity of every individual. Envisioning a future where everyone has timely access to the right treatment requires groundbreaking scientific discoveries as much as a concerted effort to ensure these innovations are rapidly integrated into clinical practice. This necessitates not only streamlining how research findings are communicated to practitioners but also advocating for policies that facilitate the swift adoption of evidence-based innovations. Central to our efforts is the principle that healthcare innovation must serve the best interest of patients, which means working with them: ‘Nothing for us, without us’.

## Birth of a strong European Health Union: a look back at the last 5 years

KyriakidesStella
European Commissioner for Health and Food Safety

Health policy has been at the heart of the Commission’s work for the past 5 years. In many ways, it has defined it. When I was appointed as European Commissioner for Health and Food Safety in December 2019, I could never have imagined the journey upon which we were about to embark. The task I was entrusted with set an ambitious and promising health agenda for the years ahead. Just a few weeks later, our focus was forcefully redirected towards largest public health crisis in a century. The COVID-19 pandemic tested our health systems, our Union and each of us to the limit. And yet, it is with great pride that we can now say that we rose to the challenge of that crisis and emerged much stronger as a Health Union.

Without a doubt, the COVID-19 pandemic was a period of great suffering and hardship across Europe and the world. However, it was also a period that brought out the very best of European values: resilience, cooperation and solidarity, helping us to deliver for our citizens under the most testing of circumstances.

This was never clearer than with the EU Vaccine Strategy, through which lifesaving COVID vaccines were made available to all citizens and Member States at the same time. This was an historic moment and it showed that this was an Union that delivers for all of its citizens, equally. It was also the dawn of a new day for cooperation in the arena of health.

Harnessing this spirt of collaboration, since November 2020, the EU and its Member States began the journey of creating a strong European Health Union. The first task was to ensure that the EU is ready to better anticipate and address emerging health threats. We now have a stronger cooperation framework on serious cross-border threats. We also strengthened the mandates of the European Medicines Agency and the European Centre for Disease Prevention and Control, and set up the new Health Emergency Preparedness and Response Authority (HERA), which is already delivering concrete results. Therefore, in many ways, better health security is a hallmark of our Health Union.

In addition to reinforcing our common EU preparedness, a strong European Health Union is one in which every citizen can access the best standards of treatment and care. This is why all our actions aim to make our health systems stronger, more resilient, and flexible enough to meet the challenges of the 21st century.

Our proposed reform of the EU pharmaceutical legislation after 20 years will create a true Single Market for medicines, ensuring universal access for to affordable medicines for patients and to promote a thriving pharmaceutical industry. We are also taking concerted and coordinated action at EU level to prevent shortages of essential medicines.

The bold proposal for the European Health Data Space, which has been agreed upon with Member States and the European Parliament, will empower people to fully access and control their health data, safeguarded by the strictest of data privacy rules. This same data will help scientists, academics and industry to develop the next groundbreaking and life-changing medicines.

Europe’s Beating Cancer Plan is one of the world’s most comprehensive blueprints for action against cancer, with EUR 4 billion in funding for concrete projects to tackle this disease at every stage. The plan and the life-changing improvements in access to diagnosis, treatment and care it is delivering for patients and their families are a source of hope for them, to show them that they are not alone on their journey.

Likewise, I believe that our work on mental health during this mandate will stand the test of time. Our Comprehensive Approach on Mental Health put mental and physical health on a par, breaking down stigmas and barriers with 20 flagship actions and over EUR 1.2 billion in funding.

In our interconnected world, it is very clear that the greatest challenges facing us—from climate change to the increasing threat of antimicrobial resistance—are shared ones. They do not respect borders and we can only tackle them if we work together with international partners a One Health approach. These principles lie at the heart of our EU Global Health Strategy, our vision for leadership and cooperation on global health for the next 10 years.

Over the past 5 years, the world has changed irrevocably—and it will continue to change, bringing new challenges and complexities. Yet, as the mandate draws to a close, I firmly believe that we leave the EU better prepared for what is to come. We have forged a stronger European Health Union, strengthened our resilience against future threats and left the legacy of a healthier, safer Europe for all.

## A global pandemic agreement: a once-in-a-generation opportunity

KlugeHans Henri
WHO/Europe Regional Director

In a world emerging from the ravages of the COVID-19 pandemic, the need to strengthen global health emergency preparedness is undeniable.

The so-called Pandemic Agreement being negotiated ahead of the 77th World Health Assembly in May 2024 aims to be a legally binding instrument under the WHO Constitution to strengthen prevention, preparedness and response.

In 2021, Member States agreed to the process of negotiating an agreement, recognizing the pandemic’s devastating impacts, acknowledging the gaps in coordination and response, and driven by the urgent need to be better prepared to respond to future similar crises.

To this end, countries have continually come together through the purposefully created Intergovernmental Negotiating Body to ensure that the development of the Agreement is Member State led, representing a commitment to jointly address shared health threats.

For the world as a whole, the Agreement is crucial. It ensures that ‘all’ countries, regardless of economic status, can effectively prevent, prepare for and respond to pandemics. This global solidarity is vital for safeguarding public health, preventing catastrophic consequences and ensuring that we break the cycle of panic and neglect by addressing gaps in the global health security architecture.

The current contents of the draft Agreement, albeit amid ongoing negotiations, seek to address the inequities in pandemic response, such as uneven vaccine distribution, and aim to strengthen global health infrastructure, including that of the WHO.

More specifically, key elements being negotiated include operationalizing equitable access to vaccines, therapeutics and diagnostics through the creation of a Pathogen Access and Benefit Sharing mechanism and meaningful transfer of technology. This ensures that those at greatest risk from an emerging infection can access affordable countermeasures, while also ensuring availability of personal protective equipment to keep the health workforce safe.

To ensure that any pathogens that do emerge are stopped in their tracks, the Agreement further seeks to strengthen global surveillance, in particular by strengthening collaboration between sectors acting in the human, animal and environmental interface, via the so called ‘One Health’ approach.

Importantly, this is complemented by a commitment to transparency in the sharing of critical epidemiological, virological, genomic, clinical and other health information between Member States.

Achieving all of this requires resources and—not least—effective governance. That is why key articles of the Agreement address mechanisms for financing preparedness and response, including within and between Member States and non-State actors, as well as enhanced plans for coordination. Moreover, the Agreement seeks ways forward to ensure compliance to make sure governments place preparedness and response at the heart of their budgeting and political priorities.

The Agreement will *not* require any government to cede national sovereignty to the WHO. On the contrary, it reinforces Member States’ responsibility to safeguard their populations—and governments are left fully able to decide how best to respond to a crisis, while offered guidance based on WHO best practices. Neither will the Agreement impose vaccine mandates on any individual, community or country; nor will it give WHO the authority to enforce lockdowns, dictate medical treatment or undertake surveillance. Drafts of the negotiation text make it clear that WHO will never have such a role.

WHO—founded 75 years ago—is tasked as the directing, coordinating authority in international health, and it seeks to perform this to the best of its ability through working with Member States. A key lesson that COVID-19 taught us was that in an increasingly interconnected world, multilateralism is essential for addressing complex challenges. Multilateralism such as that exhibited by the ongoing Intergovernmental Negotiating Body Processes enhances WHO’s ability to serve its mandate by facilitating collaboration and pooling resources.

At the same time, it will be important to align discussions on the Agreement with the ongoing process of International Health Regulations amendments led through the Working Group on the IHR whose mandate is to work on consideration of proposed targeted amendments by the 77th World Health Assembly this year. More than 90 WHO Member States have put forward over 300 proposed amendments to make the IHR fit for purpose. Outcomes from these processes will be contextualized through the upcoming regional strategy to strengthen health emergency preparedness, response and resilience in the WHO European Region 2024–2029, titled Preparedness 2.0.

For WHO/Europe, the Agreement complements and feeds into Preparedness 2.0, our new regional strategy and action plan on the strengthening of health emergency preparedness, response and resilience to better support our 53 Member States. This, in turn, speaks to the work of the Pan-European Network on Disease Control, launched by WHO/Europe in April together with the UK Health Security Agency, whose creation was recommended by the Pan-European Commission on Health and Sustainable Development convened at the height of the COVID-19 pandemic by WHO/Europe and led by Professor Mario Monti.

What is abundantly clear by now is that collective action is crucial for building a safer future. For the Pan-European region and beyond, these ongoing processes offer a once-in-a-generation opportunity to work together, and to show that we can, and will, do better when the next global public health threat arises.

## Sailing the waves of European public health: exploring a sea of innovation

MexiaRicardo
Chair of the 17th EPH Conference, Lisbon 2024
BarnhoornFloris
Deputy Director EPH Conference

The 17th European Public Health (EPH) Conference, Lisbon, Portugal, 12–15 November 2024, is themed ‘Sailing the waves of European public health: exploring a sea of innovation’. This theme symbolizes our collective endeavour in the public health domain, mirroring the spirit of ancient explorers who ventured into unknown waters. Considering the European Commission has named Lisbon as this year’s European Capital of Innovation, we will be at the perfect setting.

The Conference includes an extensive programme, featuring the traditional 16 tracks designed to broaden the scope of public health knowledge and foster numerous networking opportunities. Additionally, we are planning a diverse array of pre-conferences, details of which are forthcoming.

We are excited to announce the ‘*Conferência de Saúde Pública da Lusofonia*’, on 11 and 12 November 2024, specifically targeting the global Lusophone community. This initiative recognizes and celebrates the unique cultural ties among Portuguese-speaking countries, aiming to foster a collaborative environment for public health innovation and discussion. The Lusofonia Public Health Conference aims to promote debate and reflection around strengthening cooperation in public health between the nine Member States of the Community of Portuguese Speaking Countries (CPLP) within the scope of the objectives and goals of the CPLP Strategic Health Cooperation Plan 2023–2027 (PECS/CPLP 2023–2027).

Thematic areas include National Health Systems, Training and Development of the Health Workforce, Health Information and Communication, Monitoring of Sustainable Development Goals, Public Health Emergency Preparedness and Research and Bioethics in Health. These themes will be addressed in plenary sessions and round tables, spread out over the two-day conference. The programme also includes oral presentations for which abstracts are cordially invited. Deadline for abstract submission is 31 May 2024. The abstracts selected for presentation in free communication and poster formats at the Lusofonia Public Health Conference will be published in the *Anais do IHMT*.

The conference language will be Portuguese. Simultaneous translation into English will be provided. Detailed information here.

Registration, with reduced fees for delegates from the Portuguese speaking countries, is through the EPH Conference registration panel.

The Lusophone Public Health Conference is organized by EPH Conference, the European Public Health Association (EUPHA), the Portuguese Association for Public Health (APPSP) and the National Association of Public Health Doctors (ANMSP). The conference is held at the Lisbon Congress Center, Portugal.

Furthermore, in anticipation of the conference, we are excited to announce the ‘Sailing to Lisbon 2024’ Webinar series, serving as a prelude to the conference, offering a platform for preliminary discussions, sharing of ideas and networking among public health professionals. This webinar series is not just a build-up to the conference but an integral part of our journey towards innovation in public health. It offers an opportunity for continuous learning and engagement with the themes that will define the future of public health in Europe and beyond. And there is even an added incentive for participation, as there is a quiz at the end of each webinar, awarding prizes that range from free EPH Conference registrations to entrances for the Conference Dinner and Dance event.

The 17th EPH Conference in Lisbon invites public health professionals and students to navigate the vast ocean of possibilities within our field, charting a course towards a future where innovation and collaboration lead the way in addressing the complex health challenges of our time.

Keep updated on all the details at the conference website: www.ephconference.eu

See you in Lisbon!

